# Modified McKeown Minimally Invasive Esophagectomy for Esophageal Cancer: A 5-Year Retrospective Study of 142 Patients in a Single Institution

**DOI:** 10.1371/journal.pone.0082428

**Published:** 2013-12-20

**Authors:** Baofu Chen, Bo Zhang, Chengchu Zhu, Zhongrui Ye, Chunguo Wang, Dehua Ma, Minhua Ye, Min Kong, Jiang Jin, Jiang Lin, Chunlei Wu, Zheng Wang, Jiahong Ye, Jian Zhang, Quanteng Hu

**Affiliations:** Department of Thoracic Surgery, Taizhou Hospital, Wenzhou Medical College, Linhai, Zhejiang, China; Virginia Commonwealth University School of Medicine, United States of America

## Abstract

**Background:**

To achieve decreased invasiveness and lower morbidity, minimally invasive esophagectomy (MIE) was introduced in 1997 for localized esophageal cancer. The combined thoracoscopic-laparoscopic esophagectomy (left neck anastomosis, defined as the McKeown MIE procedure) has been performed since 2007 at our institution. From 2007 to 2011, our institution subsequently evolved as a high-volume MIE center in China. We aim to share our experience with MIE, and have evaluated the outcomes of 142 patients.

**Methods:**

We retrospectively reviewed 142 consecutive patients who had presented with esophageal cancer undergoing McKeown MIE from July 2007 to December 2011. The procedure, surgical outcomes, disease-free and overall survival of these cases were assessed.

**Results:**

The average total procedure time was 270.5±28.1 min. The median operation time for thoracoscopy was 81.5±14.6 min and for laparoscopy was 63.8±9.1 min. The average blood loss associated with thoracoscopy was 123.8±39.2 ml, and for laparoscopic procedures was 49.9±14.3 ml. The median number of lymph nodes retrieved was 22.8. The 30 day mortality rate was 0.7%. Major surgical complications occurred in 24.6% and major non-surgical complications occurred in 18.3% of these patients. The median DFS and OS were 36.0±2.6 months and 43.0±3.4 months respectively.

**Conclusions:**

Surgical and oncological outcomes following McKeown MIE for esophageal cancer were acceptable and comparable with those of open-McKeown esophagectomy. The procedure was both feasible and safe – properties that can be consolidated by experience.

## Introduction

Surgical resection remains the primary treatment for localized esophageal cancer. It increases the probability of cure and alleviates the symptoms of dysphagia as compared with non- operative approaches. However, traditional open esophagectomy carries significantly high risks of operative morbidity and mortality. The morbidity rates associated with esophagectomy vary between 30% and 80% across different centers [1]–[3]. Medicare data from the United States of America, has shown that the mortality rates following esophagectomy ranged from approximately 8% in high-volume centers to about 23% in centers performing a low volume of cases for this complex operation [4].

Regardless of the surgical approach, the aim of esophageal surgery is to obtain acceptable outcomes and to decrease procedural-related morbidity and mortality. Advances in surgical techniques and equipment have made minimally invasive esophagectomy (MIE) more popular and widely acceptable since the 1990s [5]–[7]. The potential advantages of MIE include reduced trauma, less complex post-operative recovery, and fewer incidences of wound and pulmonary complications [8]–[9].

During the past two decades, MIE has been accepted as an alternative treatment approach for esophageal cancer around the world. Subsequently, various minimally invasive surgical (MIS) approaches for treating esophageal cancer have been reported since 1992. However, many of these studies described minimally invasive hybrid approaches, including thoracoscopic-laparotomy or laparoscopy-thoracotomy. Few studies exist where a small group of patients presenting with esophageal cancer have been treated with a combined thoracoscopic and laparoscopic surgical approach. Additionally, for potentially less invasive and lower morbidity outcomes, simple video-assisted thoracoscopic esophagectomy was introduced at Taizhou hospital of the Wenzhou Medical College in 1997 for the treatment of esophageal cancer. Subsequently in 2007, our institution implemented laparoscopic gastric mobilization, which was developed from the McKeown MIE procedure. Our institution subsequently evolved into a high-volume MIS treatment center for esophageal cancer.

In the present study, we report on a retrospective study and surgical outcomes that were obtained by treating 142 consecutive patients with the McKeown MIE procedure. The aim of this analysis was to evaluate the technical feasibility, and the surgical and oncological safety of this procedure in a larger group of patients from the Eastern parts of China.

## Patients and Methods

### Patients and Pre-operative Evaluation

Prior written informed consent was obtained from the patients and the study received ethics board approval at Taizhou Hospital, Wenzhou Medical College. A prospective database with perioperative variables, survival and recurrence data of patients undergoing esophagectomy was established in 1997 and maintained at our institution. Additionally, written consent was given by the patients for their information to be stored in the hospital database for future research. We retrospectively analyzed 142 consecutive patients who presented with esophageal cancer, and underwent McKeown MIE at the Department of Thoracic Surgery, Taizhou Hospital, between July 2007 and December 2011.

All patients were diagnosed as esophageal cancer by pathological criteria using upper endoscopy and biopsy specimen analysis. Simultaneously, every patient had a comprehensive pre-operative evaluation consisting of clinical presentation, physical examination, pulmonary function tests, electrocardiography, cardiac echocardiography, endoscopic ultrasound (EUS), contrast-enhanced computed tomography (CT) scans of the chest and abdomen, positron emission tomography (PET) scans, and barium meal assessment. Patients with pre-operative stage T>3, or gastroesophageal junction cancer, or who had received neoadjuvant chemoradiotherapy, or had received hybrid MIE were all excluded from this study.

### Surgical Approach

The thoracoscopic portion of the operation was first performed to evaluate the features of the tumor. The thoracoscopic procedure for esophageal mobilization and mediastinal lymphadenectomy has been previously described [10], [11].

**Figure 1:**
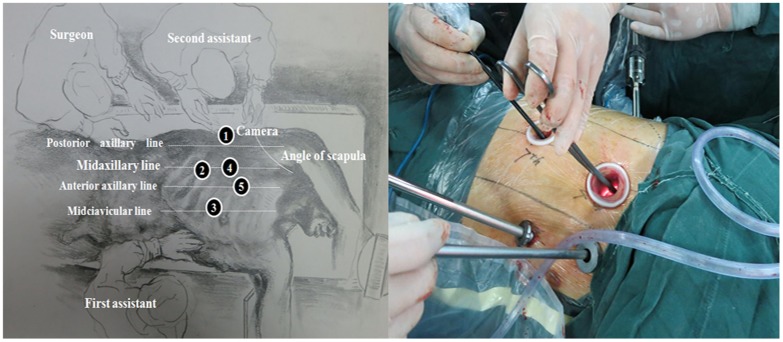
Patient positioning and trocar position for thoracoscopic portion.

**Figure 2:**
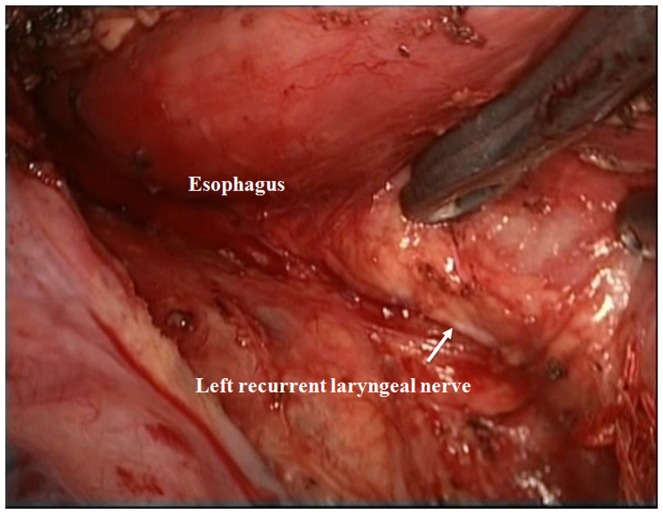
Thoracoscopic exposure of the left recurrent laryngeal nerve and lymph node dissection.

**Figure 3:**
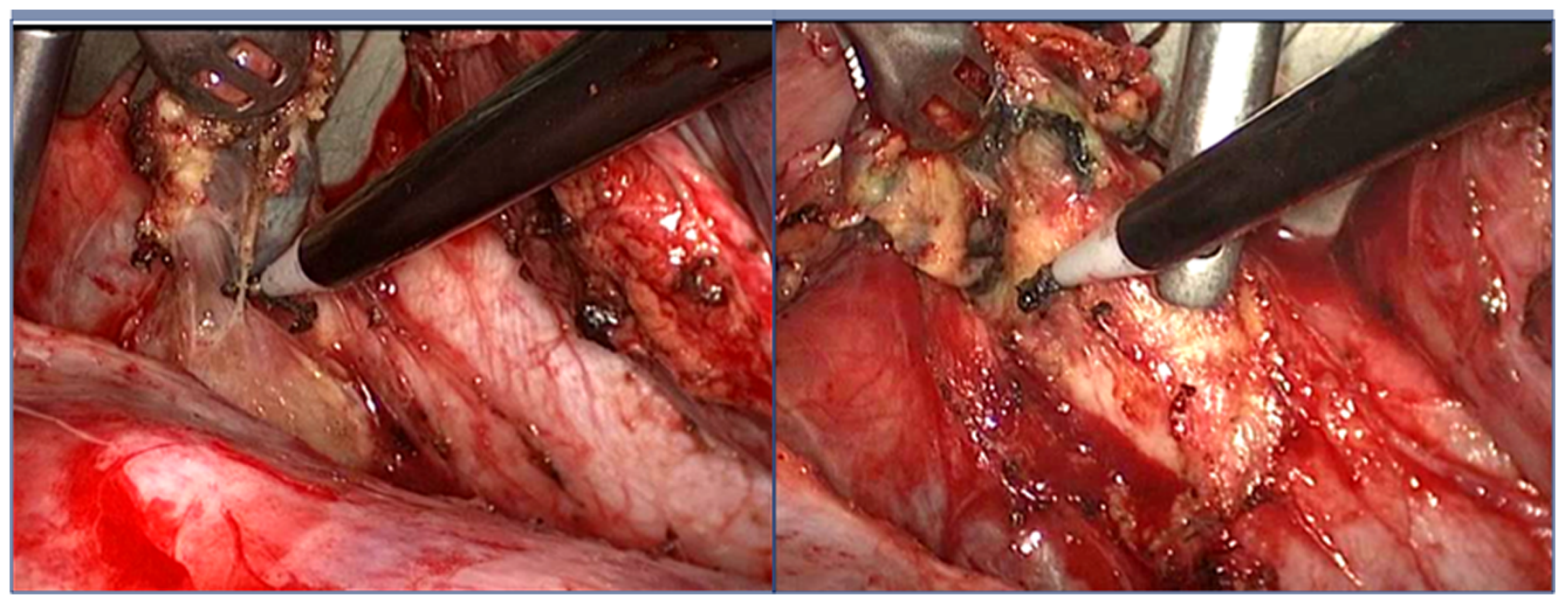
Thoracoscopic subcarinal lymph node dissection.

After general anesthesia and endotracheal intubation, the patient was placed in the left lateral decubitus position. Next, four 10 mm trocars, and one 5 mm trocar were placed as shown in **Figure 1**. The surgeon and thoracoscopic technician stood facing the patient's back and opposite the first assistant. First, the right recurrent laryngeal nerve lymph nodes were dissected and the mediastinal pleura were exposed at the level of the inferior pulmonary vein to commence esophageal mobilization. The azygos venous arcade was clipped to expose the esophagus using hem-o-lock ligating clips. After mobilizing the thoracic esophagus from the hiatus to the thoracic inlet, an aggressive mediastinal regional lymphadenectomy was carried out (i.e., the left recurrent laryngeal nerve **[** **Fig. 2** **]**, paraesophageal, subcarinal **[** **Fig. 3** **]**, paratracheal and supradiaphragmatic lymph nodes). If necessary, the thoracic duct was mobilized and ligated between the azygos vein and the descending aorta at the level of the 10th through 12th thoracic vertebra. Finally, a single 26-F chest canula was inserted and the chest incision was sutured, thus completing the thoracoscopic procedure.

**Figure 4:**
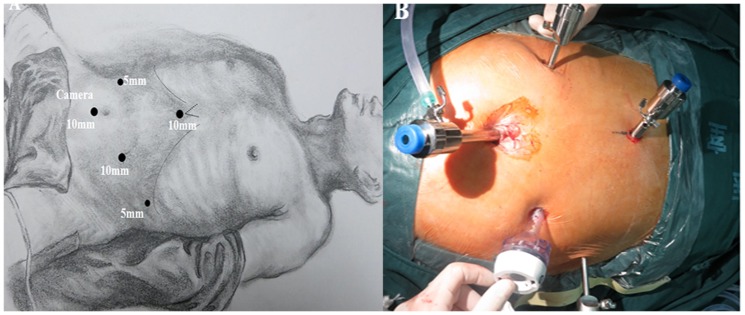
Patient positioning and trocar position for laparoscopic portion.

After completing the thoracoscopic procedure, the patient was rotated to a lithotomic position, with the neck extended and turned toward the right. The surgeon stood between the patient's thighs, with surgical assistance positioned to the right (camera) and the left side of the patient. Pneumoperitoneum was established with 12–15 mmHg with CO_2_, following which, five abdominal trocars were inserted in the form of a “V”-shaped distribution, and a 5 mm trocar was then inserted in the subxiphoideus as depicted in **Figure 4**.

**Figure 5:**
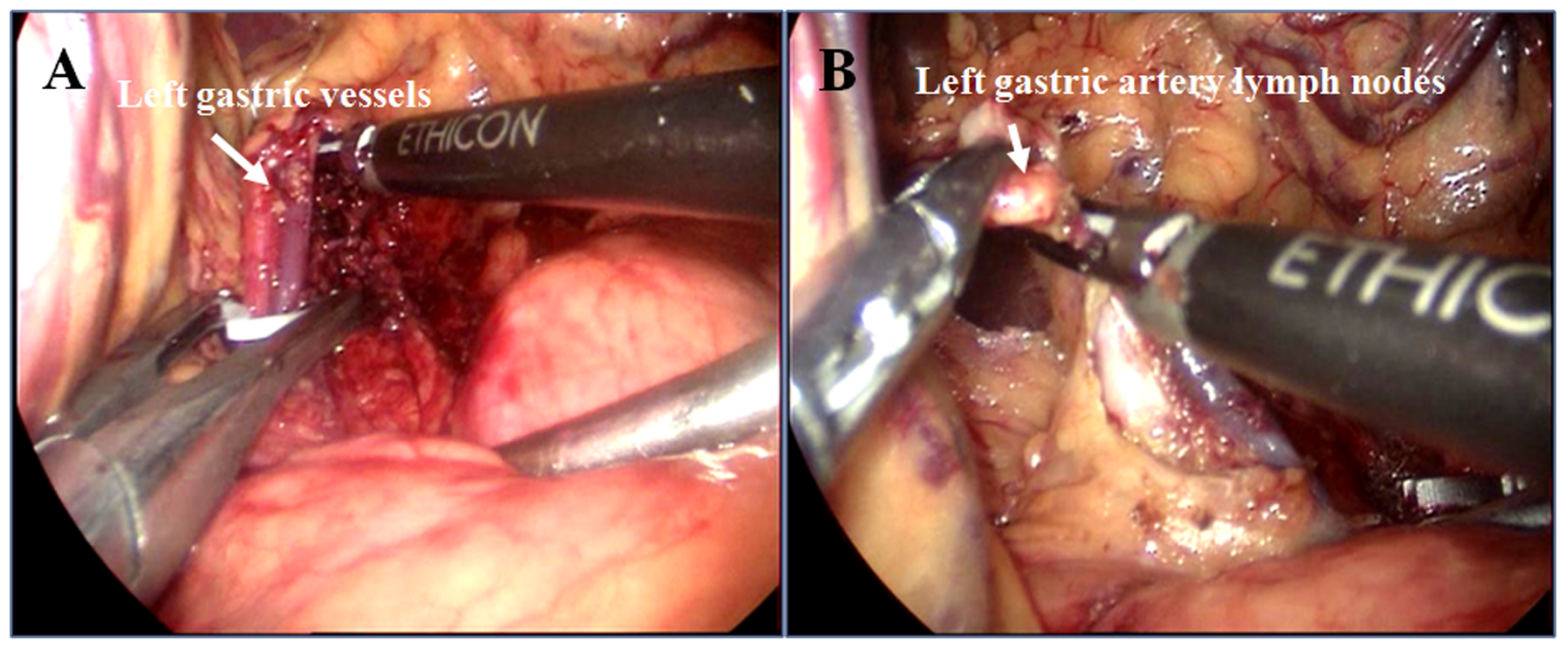
Laparoscopic mobilization and division of the left gastric vein and artery (A) and lymph node dissection (B).

The entire greater curvature of the stomach was first mobilized by dividing the gastrocolic ligament and then separating the greater omentum using ultrasonic shears, followed by division of the short gastric vessels and disarticulation of the gastrosplenic ligament to reveal the left crus of the diaphragm. Next, the gastrohepatic ligament and lesser omentum were divided to expose the right crus of the diaphragm. The left gastric vessels were dissected and divided using ultrasonic shears after having been isolated using the hem-o-lock ligating clips **(** **Fig. 5** **)**. Subsequently, abdominal lymphadenectomy was performed (i.e., the celiac trunk, left gastric vessels, cardiac, and greater and lesser curvatures of the stomach). Finally, the right and left crura of the diaphragm were dissected and the esophageal hiatus was widened, linking the abdominal cavity to the mediastinum.

An approximate 5 cm oblique incision was made over the anterior border of the left sternocleidomastoid muscle. The neck esophagus was mobilized to permit communication with the right chest, taking care to preserve the left recurrent laryngeal nerve. Simultaneously, left neck lymphadenectomy was performed. The neck esophagus was manually raised and transected, after which the distal end was connected to the thick rubber tube. Immediately following this procedure, the subxiphoideus interspace port was enlarged to 5 cm to construct an approximate 3–5 cm diameter gastric conduit to remove the specimen. Ultimately, the gastric conduit was pulled up to the left neck through the posterior mediastinum or retrosternal tunnel, assisted by the rubber tube to enable esophagogastric hand-sewn anastomosis.

**Figure 6:**
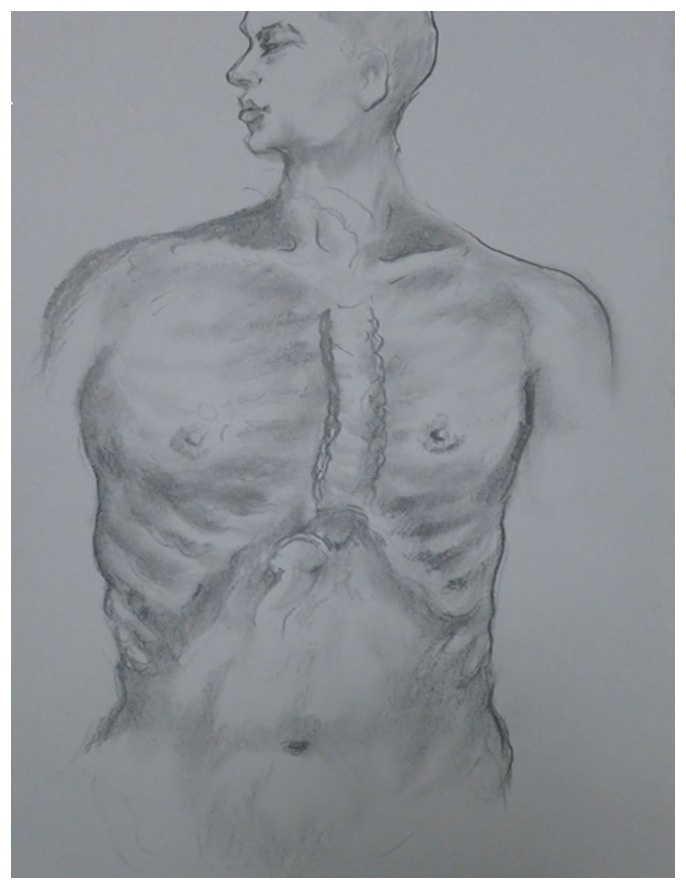
Holistic and complete view of the McKeown minimally invasive esophagectomy procedure.

Additionally, in a few patients, the gastric conduit was completed by using the endoscopic stapler in the absence of using a small incision under the xiphoid. Pyloroplasty and feeding jejunostomy were not performed in any of the patients. The holistic and complete view of the McKeown MIE procedure is shown in **Figure 6**.

### Post-operative Care

After the operation, most of the patients were extubated and transferred to the general ward. Additionally, few patients were admitted to the ICU and placed on mechanical ventilation because of not weaning from the respirator. Patient-controlled analgesia is routinely used. Additionally, all patients were given parenteral nutrition through the jugular vein or enteral nutrition through a nasogastric tube. The chest tube and nasogastric tube were removed after beginning an oral soft diet, and the patient was then discharged. Almost all of the patients, who had presented with lymph node metastasis or T3, received post-operative chemotherapy.

### Oncological Assessment, Follow-up and Survival

Post-operative pathological staging was defined according to the TNM classification (6^th^ ed.) of the International Union Against Cancer. Patients were seen for follow-up at 3-month intervals during the first year and every 6 months thereafter. Simultaneously, we conducted a telephone follow-up. Disease-free survival (DFS) was defined as the interval between the surgical procedure and the first evidence of tumor progression or death. Overall survival (OS) was the interval from the day of surgery until death.

### Statistical Analysis

Descriptive statistics (mean ± SD) were provided in this report. Survival was analyzed by Kaplan-Meier method for the whole patient population and compared using the log-rank test. All statistical analyses were performed with a dedicated analysis tool (SPSS 17.0 statistical software package; SPSS, Chicago, Il, USA).

## Results

### Patient Demographics and Clinicopathological Characteristics

The clinical and pathological characteristics of the patients are listed in **Table 1**. There were 91 men and 51 women with a mean age of 60.5±8.2 years (range, 47–79 years). For 81 patients (57.0%), the tumor was located in the middle third of the esophagus, whereas for 42 patients, the tumor was located in the lower third. In 19 patients, the tumor was located in the upper third of the esophagus. Of those, 50 patients had co-morbid conditions (**Table 1**). Additionally, 131 patients had squamous cell carcinoma, whereas the remaining cases had adenocarcinoma or presented with other types of tumor. Most patients had a T2 tumor (n = 56), whereas 50 patients had a T1 tumor and 36 patients had a T3 tumor. In 93 patients, the tumor length was less than 3 cm, whereas in 41 cases the tumor length was in the range of 3 cm–5 cm, only 8 patients had a tumor length of more than 5 cm. Of all the cases, 44 patients (31%) were examined for lymph node metastasis.

**Table 1:** Demographics and clinicopathological parameters (n = 142).

Variables	N = 142	
Demography	Age (mean ± SD)	60.5±8.2
	Male: n (%)	91 (64.1)
	Female: n (%)	51 (35.9)
ASA-Score	ASA-1: n (%)	81 (57.0)
	ASA-2: n (%)	53 (37.3)
	ASA-3: n (%)	8 (5.6)
Comorbidity	Hypertension: n (%)	17 (12.0)
	Diabetes: n (%))	14 (9.9)
	COPD: n (%)	5 (3.5)
	Liver cirrhosis: n (%)	3 (2.1)
	Previous chest surgery: n (%)	4 (2.8)
	Previous abdominal surgery: n (%)	7 (4.9)
Location of lesion	Upper third: n (%)	19 (13.4)
	Middle third: n (%)	81 (57.0)
	Lower third: n (%)	42 (29.6)
Histological type	Squamous carcinoma: n (%)	131 (92.3)
	Adenocarcinoma or other: n (%)	11 (7.7)
Depth of tumor invasion	Tis∼1: n (%)	50 (35.2)
	T2: n (%)	56 (39.4)
	T3: n (%)	36 (25.3)
Tumor size	≤3 cm: n (%)	93 (65.5)
	3 cm∼5 cm: n (%)	41 (28.9)
	≥5 cm: n (%)	8 (5.6)
Lymphatic metastasis	Nx-0: n (%)	98 (69.0)
	N1: n (%)	44 (31.0)

**Abbreviations**: ASA-score: American Society of Anesthesiologists score. COPD: chronic obstructive pulmonary disease.

### Surgical Outcomes

A relatively consistent and experienced team of surgeons, who worked in conjunction with a thoracic surgeon, performed all procedures. A total of 142 patients successfully underwent McKeown MIE as described above and between July 2007 through December 2011, with no intra-operative death or conversion to a traditional open procedure. The surgical procedure and outcomes are listed in **Table 2**. Gastric conduit was pulled up to neck to reconstruct the upper digestive tract in all cases. For reconstruction, the esophageal bed route was used in 104 cases, whereas the retrosternal route was selected in 38 cases. The average total procedure time was 270.5±28.1 min (range, 196–320 min). The median operation time for thoracoscopy was 81.5±14.6 min (range, 60–130 min) and for laparoscopy it was 63.8±9.1 min (range, 40–90 min). The mean blood loss associated with thoracoscopy was 123.8±39.2 ml (range, 60–310 ml), and that of the laparoscopic procedures was 49.9±14.3 ml (range, 30–100 ml). The median number of lymph nodes retrieved was 22.8 (range, 5–48). The average number of harvested mediastinal lymph nodes was 13.5 (range, 3–30), and that of the harvested abdominal nodes was 8.3 (range, 2–18). The median post-operative duration of hospital stay was 12.2 days (range, 9–45 days). The 30-day mortality rate was 0.7% (n = 1).

**Table 2:** Operative and post-operative parameters.

Variables	N = 142	
Blood loss (mL)	Thoracoscopy (mean ± SD)	123.8±39.2
	Laparoscopy (mean ± SD)	49.9±14.3
Operation time (min)	Total: average (mean ± SD)	270.5±28.1
	Thoracoscopy (mean ± SD)	81.5±14.6
	Laparoscopy (mean ± SD)	63.8±9.1
R0 resection	n (%)	142 (100.0)
Anastomosis	Left neck anastomosis: n (%)	142 (100.0)
	Hand-sewing: n (%)	142 (100.0)
Upper gastrointestinal reconstruction	Gastric conduit: n (%)	142 (100.0)
	Esophageal bed route: n (%)	104 (73.2)
	Retrosternal tunnel rout: n (%)	38 (26.8)
ICU stay (days)	Average (range)	1 (0–5)
Post-operative hospital stay (days)	Average (range)	12.2 (9–45)
No. of lymph nodes harvested	Total: average (range)	22.8 (5–48)
	Mediastinal: average (range)	13.5 (3–30)
	Abdominal: average (range)	8.3 (2–18)
	Left cervix: average (range)	1 (0–6)

### Complications

Detailed post-operative complications are shown in **Table 3**. Major surgical complications occurred in 35 patients (or 24.6%). There was no evidence of post-operative hemorrhage. Vocal cord palsy developed in 8 patients (5.7%) who recovered within 3 to 6 weeks. Anastomotic leak and gastric necrosis were detected in 10 patients (7.0%) in whom 3 patients required a follow-up operation, and the remaining patients were managed conservatively through nutritional support. One patient (0.7%) developed a bronchial fistula and was treated with a tracheal stent, but eventually died of multiple organ failure 28 days after surgery. Of 5 patients (3.5%) who were diagnosed with chylothorax or chylous ascites, 3 received thoracoscopic thoracic duct ligation. In 11 (7.7%) patients, anastomotic stenosis was detected and treated by gastroscopic dilatation or esophageal stenting. Major non-surgical complications occurred in 18.3% of the patients, including 13 (9.2%) patients with respiratory pneumonia, 3 (2.1%) with respiratory failure, 4 (2.8%) with arrhythmia, and 6 (4.2%) with delayed gastric emptying, which were managed and treated conservatively.

**Table 3:** Post-operative complications.

Variables	N = 142	N (%)
**Major surgical complications**	Total	35 (24.6)
	Vocal cord palsy	8 (5.6)
	Anastomotic leak	9 (6.3)
	Tracheo-bronchial injury	1 (0.7)
	Gastric necrosis	1 (0.7)
	Anastomotic stenosis	11 (7.7)
	Chylous ascites	1 (0.7)
	Chylothorax	4 (2.8)
**Major non-surgical morbidity**	Total	26 (18.3)
	Respiratory Pneumonia	13 (9.2)
	Respiratory failure	3 (2.1)
	Arrhythmias	4 (2.8)
	Delayed gastric emptying	6 (4.2)
**In-hospital/30-days mortality**		1 (0.7)

### Follow-up and Survival

**Figure 7:**
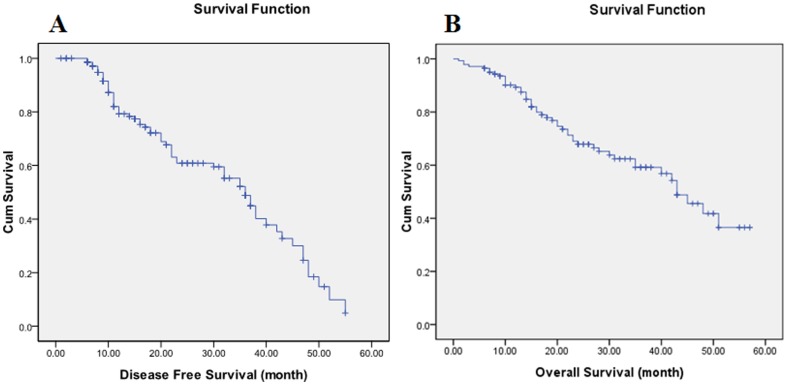
Disease-free survival (A) and overall survival (B) of patients presenting with esophageal cancer who received the McKeown minimally invasive esophagectomy procedure.

The median follow-up time was 26 months (range = 6–57). The median OS was 43.0±3.4 months, with 89% showing 1-year OS, and 67% showing 2-year OS. The median DFS was determined to be 36.0±2.6 months, with 79% of patients showing 1-year-DFS and 61% of patients showing 2-year-DFS [Fig. 7].

## Discussion

Esophagectomy is a complex and technically challenging surgical procedure associated with high mortality and morbidity. However, surgical resection is the primary treatment for resectable esophageal cancers, and in order to reduce the mortality and morbidity associated with open operations, various minimally invasive surgical approaches for esophagectomy have been increasingly applied and reported [5]–[7].

The early application of MIE was thoracoscopic esophagectomy combined with laparotomy [5]. Although a reduction in patient trauma and sensation of pain was achieved, there was no conclusive benefit between those patients who underwent thoracoscopic surgery and those that underwent thoracotomy. Subsequently, laparoscopic transhiatal esophagectomy in combination with neck anastomosis was reported [12]. However, this procedure made both mediastinal lymphadenectomy and the resection of upper and middle esophageal cancer very challenging owing to the limitations of available working space [13]. Therefore, Luketich and his colleagues adopted the combined thoracoscopic-laparoscopic esophagectomy approach and believed that MIE with stage-specific survival was equivalent to previously published open series cases [9], [14]. At the present time, minimally invasive “Ivor Lewis” esophagectomy, which is performed in some centers, is thought to reduce the morbidity associated with RLN dysfunction [15]–[17].

For a less invasive approach, and to achieve lower morbidity, thoracoscopic esophagectomy combined with laparotomy was performed at our institution in 1997. While we adopted laparoscopic gastric mobilization and abdominal lymphadenectomy to esophageal cancer in July 2007, the McKeown MIE procedure was subsequently developed as a high-volume MIS treatment center for esophageal cancer in China. Approximately 450 patients with esophageal cancer underwent different three-incision MIS, of these, 142 patients received the McKeown MIE procedure until December 2011. The outcomes of McKeown MIE at our institution are comparable to those of transthoracic and transhiatal approaches, and are an improvement over MIE previously reported [11].

In our procedure, the shorter operation time and decreased blood loss were due to anatomic familiarity, extensive experience, and improved visualization of the endoscopic equipment. The two-field lymphadenectomy is performed as an open surgery. The average number of harvested mediastinal nodes was 13.5, and that of the harvested abdominal nodes was 8.3. There were no differences in the lymph nodes dissected between MIE and the open procedure at our center [10]. This was consistent with the current literature [17]–[18]. Additionally, with a mean follow-up of 26 months, our Kaplan-Meier 2-year survival rate was 67%, which was similar to that reported for open esophagectomy [19].

The selection of surgical cases is very important, especially during the initial stages of the learning curve. Eligible patients who met the inclusion criteria, including pre-operative stage T≤3, a tumor length ≤5 cm, and with no lymph node involvement, were only considered for MIE. A history of abdominal surgery is not an absolute contra-indication; certain patients who had undergone laparoscopic cholecystectomy or appendectomy were eligible for laparoscopic surgery.

The potential benefits of McKeown MIE are a more proximal resection margin and improved lymph node dissection. However, these procedures are associated with a higher morbidity, which is partly due to RLN injury and anastomotic leak, and stricture as reported by Luketich. For this reason, Luketich *et al.* altered their approach by switching from McKeown MIE to Ivor Lewis MIE with intrathoracic anastomosis. They then found a lower incidence of RLN and anastomotic complications [16]–[17].

The majority of tumors that we identify are located in the middle esophagus, which needs an adequate proximal esophageal resection margin, whereas in Western countries, such tumors tend to be located in the distal esophagus and gastroesophageal junction. In addition, the principal pathological types that we encounter are squamous cell carcinoma, which easily appear along with lymph node metastases. An aggressive 2 or 3-field lymphadenectomy after esophagectomy can improve patient survival. Thus, the McKeown MIE approach is performed as a routine procedure at our institution, which is more in line with the oncological principles and is suitable for esophageal cancer in China. Although anastomotic leak rates are equivalent between both procedures, when the leaks occurred in the thoracic cavity, this presented as a much more significant concern, than those that occur in the neck. Also we believe understanding gross anatomy, and carefully performing the procedures under the much-improved thoracoscopic exposure approach can avoid RLN injury.

The thoracoscopic portion of the operation was first performed in the left lateral decubitus position to evaluate the intrathoracic esophageal tumor resectability, which is different from other medical centers at the beginning of the laparoscopic portion of the approach. The choice of position depends largely on the surgeon's preference. The prone position has an improved operative exposure, improved surgeon ergonomics, lowered anesthetic requirements, and is thus generally preferred [20]–[21]. However, the left lateral position and anatomic orientation allows the surgical team to adapt to the new procedure rapidly and enable conversion to open surgery if necessary [22]. In terms of operation time and blood loss, the prone position is not necessarily superior to the left lateral decubitus position during our procedures. From reducing patient care costs considerations, the subxiphoideus interspace port is enlarged to 5 cm to construct a gastric conduit and to remove the specimen in most cases. We would recommend this method, especially in developing countries. The route of upper gastrointestinal reconstruction for the gastric conduit after esophagectomy also remains controversial. Several studies have reported that the route was not significantly correlated with post-operative morbidity and mortality. We initially preferred the esophageal bed route because of its compatibility with human anatomy and physiology, and also utilized the retrosternal route in T3 patients receiving post-operative radiotherapy. The retrosternal route significantly decreased the impact on lung function, but was associated with a higher incidence of anastomotic leakage and pharygoesophageal swallowing dysfunction than was found for the esophageal bed route.

Considering the incidence of major surgery-related complications, MIE is acceptable and similar to open esophagectomy. Major surgical complications occurred in 24.6% of patients in our study. According to our experience, it is more important to prevent the occurrence of those adverse events rather than deal with post-operative complications only. Post-operative bleeding is the most common cause of emergency surgical exploration after esophagectomy. Although bleeding may appear in any surgically exposed areas, considering the anatomical relationship between the vessels and the esophagus, and detecting hemorrhages and incision bleeding through careful examination can effectively prevent it.

One of the most serious post-operative complications associated with surgery for esophageal cancer is anastomotic leak. Smithers *et al.* [8] compared the incidence of anastomotic leak after different esophagectomy techniques. The incidence of anastomotic leak was 8.7% for the traditional open approach, 5.5% for the thoracoscopic approach, and 4% for the thoracoscopic-laparoscopic approach. In our study, anastomotic leak occurred in 9 of 142 patients who underwent the McKeown MIE, which was similar to most other reported series of MIE approaches. Decker [23] reported a 0.8% incidence of tracheo- bronchial injury from the thoracoscopic esophagectomy with a similar incidence for open esophagectomy [24]. Similarly, this injury occurred in 1 patient in our study. Damage to the bronchus caused by thermal conductivity of the cautery hook or ultrasonic scalpel, and the erosion caused by gastric acid or other secretions following anastomotic leakage might be responsible for bronchial fistula formation.

A chylous fistula is an unwelcome complication of esophagectomy. The reported incidence is 2.4% to 11.6% after thoracoscopic esophagectomy. In our patients, the incidence was 3.5%. Most cases were managed conservatively, and the remaining patients were treated by thoracoscopic thoracic duct ligation [25]. Some published articles reported that recurrent laryngeal nerve injury was associated with a neck anastomotic and excessive lymphadenectomy [17]. In our center, the lymph nodes that are located around both sides of the recurrent laryngeal nerve are removed and neck anastomosis is typically performed without increasing the incidence of RLN injury. The RLN injury occurred in 8% of 142 patients treated. Adequate and skilled surgical operations can reduce the incidence of RLN injury. In addition, dissection of the right recurrent laryngeal nerve lymph node is recommended before mobilization of the esophagus.

Pulmonary complications remain a major non-surgical complication of the MIE procedure. Due in part to the different MIE procedures, and the lack of standard definitions of pulmonary complications, the incidence of such complications ranges from 16% to 30%. However, their incidence is relatively lower in minimally invasive surgeries than in traditional open procedures [26]. The incidence of pulmonary complications including respiratory pneumonia, and respiratory failure was 11.3% in our study. Most of these patients were cured by conservative treatment. Some severe cases, often secondary to anastomotic leakage or bronchial fistula, required more intense treatment for related complications. Arrhythmias occasionally occur, but can often be controlled by standard medication. Delayed gastric emptying can be relieved with physical therapy and medication.

Nevertheless, the strengths of our study are that it is the larger to date investigating the McKeown MIE for esophageal cancer from Eastern countries. This study is also a longer follow-up study in the evaluation of oncological outcomes of MIE. Based on our experience and an analysis of the current literature [27]–[28], the McKeown MIE was associated with lower morbidity and mortality than the conventional open esophagectomy, especially for patients with early esophageal cancer [29]–[31]. More importantly, the former ensures the transection of the esophagus with a cancer-negative margin, and shows a favorable oncological outcome compared to traditional open surgery in terms of lymph node dissection. However, MIE has a relatively longer learning curve because of the more challenging and complex manipulations required in this operation [32]. Osugi reported that 17 cases were required to acquire and practice the basic skills, and the learning curve plateaued after 35 cases [33]. MIE procedures described in the current literature were mostly performed in early esophageal cancer patients [34]. However, most patients are diagnosed in the advanced stages of esophageal carcinoma. Therefore, it is necessary to expand the scope of application of minimally invasive surgery for esophageal cancer. Minimally invasive radical esophagectomy performed after neoadjuvant chemoradiotherapy has the potential of becoming a new treatment strategy for patients with advanced esophageal carcinoma [35].

## Conclusions

We have shown that the modified McKeown MIE procedure for esophageal cancer was not only feasible and safe, but that surgical and oncological outcomes were acceptable in an experienced institution. We believe that any outstanding concerns associated with such surgical approaches and outcomes will be gradually consolidated by experience.
